# The Interplay of Obesity, Dyslipidemia and Immune Dysfunction: A Brief Overview on Pathophysiology, Animal Models, and Nutritional Modulation

**DOI:** 10.3389/fnut.2022.840209

**Published:** 2022-02-17

**Authors:** Yongbo She, Rabban Mangat, Sue Tsai, Spencer D. Proctor, Caroline Richard

**Affiliations:** ^1^Division of Human Nutrition, Department of Agricultural, Food and Nutritional Science, University of Alberta, Edmonton, AB, Canada; ^2^Metabolic and Cardiovascular Diseases Laboratory, Department of Agricultural, Food and Nutritional Science, University of Alberta, Edmonton, AB, Canada; ^3^Department of Medical Microbiology and Immunology, University of Alberta, Edmonton, AB, Canada

**Keywords:** obesity, insulin resistance, dyslipidemia, immune function, nutrition

## Abstract

Obesity has emerged as a leading global health concern. It is characterized by chronic low-grade inflammation, which impairs insulin signaling, lipid metabolism and immune function. Recent findings from animal and clinical studies have begun to elucidate the underlying mechanisms of immune dysfunction seen in the context of obesity. Here, we provide a brief review on the current understanding of the interplay between obesity, dyslipidemia and immunity. We also emphasize the advantages and shortcomings of numerous applicable research models including rodents and large animal swine that aim at unraveling the molecular basis of disease and clinical manifestations. Although there is no perfect model to answer all questions at once, they are often used to complement each other. Finally, we highlight some emerging nutritional strategies to improve immune function in the context of obesity with a particular focus on choline and foods that contains high amounts of choline.

## Introduction

Obesity is one of the most common non-communicable diseases globally and is often accompanied by several complications such as insulin resistance, diabetes, atherosclerosis, hypertension and cancer (1). According to the Obesity Canada, over 30% of Canadian adults have obesity and may need medical intervention. Elevated fasting triglycerides (TG) and low-density lipoprotein cholesterol (LDL) levels are often seen in individuals with obesity (2), which underpins a concomitant increase in cardiovascular disease (CVD) risk. On the other hand, obesity is also widely recognized as being associated with a state of chronic low-grade systemic inflammation with elevated levels of circulating pro-inflammatory cytokines (1). More recent discoveries suggested that obesity and diabetes are associated with marked changes in the immune system systemically and in metabolically relevant tissues (3), to an extent that impairs normal immune function (4). In that regard, patients with obesity-related metabolic complications (i.e. type-2 diabetes, hypertension) were also found to have an impaired immune response to infection, influenza and COVID-19 (5, 6). These findings suggest that obesity plays a key role in modulating immune function, development of metabolic complications, and related cardiometabolic diseases. In addition, obesity-related dyslipidemia may also directly modulate immune function where TG-rich lipoproteins are able to induce leukocyte activation, particularly in the postprandial state (7). The scope of the present review article is to provide a brief overview on the current understanding of immune changes in the context of obesity, applicable research models that are advancing this field of research, and potential nutritional strategies with a particular focus on dietary intake of choline and foods high in choline to improve the immune dysfunction in the context of obesity and insulin resistance.

## Obesity is Associated With Systemic Inflammation

Studies have demonstrated that subjects with obesity have elevated circulating pro-inflammatory cytokines, including tumor necrosis factor-α (TNF-α), interleukin-6 (IL-6) and C-reactive protein (CRP) (8–10). There is the notion that adipose tissue is one main contributor through producing substantial amounts of TNF-α and IL-6 (11). It is known that these pro-inflammatory cytokines impair normal insulin signaling pathways and therefore can lead to insulin resistance as well as dyslipidemia. However, the role of IL-6 *per se* may depend on the target organ. For example, IL-6 has been shown to promote glucose uptake by skeletal muscles toward improving whole body glucose homeostasis (12). Nevertheless, how inflammation is initiated in the context of obesity and further leads to a state of chronic low-grade systemic inflammation still remains to be fully elucidated.

Bornstein et al. were the first to report the involvement of macrophages in adipose tissue leading to inflammation (13). This discovery has led to an emergent hypothesis that defines the relationship between obesity-related inflammation and impaired immunity. Latter studies found that macrophage infiltration in adipose tissue is increased in obesity (14, 15). Infiltrating macrophages appear as aggregated crown-like structures and tend to shift from M2 (anti-inflammatory) to M1 (pro-inflammatory) phenotypes (1). However, recent findings have challenged the initial simplified notion of M1/M2 phenotypes in adipose tissue. The populations of adipose tissue macrophages appear to have more diverse phenotypes (i.e., metabolic activated or oxidized), with distinct surface markers activated by a wide range of stimuli such as free fatty acids or glucose (16). Mechanisms of macrophage recruitment into adipose tissue are still not fully understood, but appear to be partially due to adipose tissue expansion, leptin resistance and adipocyte death (17). M1 macrophages are pro-inflammatory and secrete substantial amounts of TNF-α and IL-6, while also secreting other pro-inflammatory mediators such as monocyte chemoattractant protein-1 (MCP-1) that can recruit additional circulating monocytes (17, 18). The understanding on macrophage infiltration is still evolving while other pro-inflammatory immune cells including CD8+ cytotoxic T cells, CD4+ T helper (Th) 1 cells, neutrophils as well as B cells (particularly the B2 subtype that promote inflammation) have also been observed during the progression of obesity in animal models (18, 19). Similarly, in mice, an accumulation of CD4+ Th cells and CD8+ cytotoxic T cells and reduction of FoxP3+ T regulatory (Treg) cells have also been observed in the small and large intestine in a diet-induced obesity (DIO) model (3). Notably, intestinal barrier integrity has also been proposed to be compromised during obesity, leading to gut-derived lipopolysaccharide (LPS) translocation that may further contribute to systemic inflammation (3).

Obesity is a heterogenous chronic disease that is influenced by multiple factors such as the environment and genetics. Obesity is also associated with a number of metabolic perturbations that can vary on an individual basis. The notion of metabolically healthy obesity (MHO) which can be defined as individuals with obesity that do not have other comorbidities such as hyperglycemia, dyslipidemia or hypertension, has drawn substantial attention in the research field (20). MHO individuals can even exhibit improved insulin sensitivity, better adipose tissue function, a favorable adipokine secretion pattern, as well as less immune cell infiltrations into adipose tissue (21). Yet, there is still a debate as to whether MHO individuals are truly healthy or if they will eventually develop metabolic perturbations associated with their obesity. In that regard, it has been shown that MHO subjects display a remarkably higher risk of developing type 2 diabetes and CVD compared to metabolically healthy lean subjects (20). Findings from a meta-analysis of 59 studies found that nearly 50% of MHO subjects developed one or more metabolic abnormalities within a 10-year window (22). We have also recently demonstrated that MHO subjects appear to have elevated plasma CRP levels (3.6 ± 3.1 mg/L), which can be categorized as low-grade systemic inflammation (4). These findings suggest that MHO individuals might be on the right trajectory for developing metabolic complications including systemic inflammation. Nevertheless, the extent to which MHO individuals have “normal” immune function when compared to healthy lean individuals remains to be investigated.

## Understanding of Immune Dysfunction in the Context of Obesity

In the clinical setting, a number of studies have confirmed the strong association between obesity, body mass index and post-surgical infections (23–25). More recently, scientists have identified that obesity is an independent risk factor for COVID-19 mortality (26). A meta-analysis of 20 cohort studies involving 28,355 patients showed that patients with obesity displayed nearly twice the risk for unfavorable outcomes related to COVID-19, along with a 50% increased risk of death (27). These emerging clinical observations support the notion that obesity may lead to immune dysfunction.

The two major arms of the immune system, namely innate and adaptive immunity, work closely together and are regulated by key messengers such as cytokines. There is an appreciable shift from anti- to pro-inflammatory immune cells in adipose tissue in the context of obesity (28), which leads to a constant exposure to higher circulating levels of pro-inflammatory cytokines. How these physiological changes will in turn impair the magnitude of their response to immune challenges remains unclear. IL-2 is a crucial cytokine produced by T cells that regulates both cell proliferation and differentiation. In 2002, Lamas et al. assessed immune responses to foreign stimuli by *ex vivo* mitogen stimulation. Cafeteria DIO Wistar rats had a significantly lower production of IL-2 from splenocytes after mitogen stimulation compared to lean controls, suggesting impaired T cell function (29). Consistent with this, Richard et al. compared immune function between subjects with obesity (stage 0; i.e., MHO) and subjects with obesity and type-2 diabetes (stage 2; i.e., with type 2 diabetes). Stage 2 patients produced significantly lower levels of IL-2, IL-6 and TNF-α from peripheral blood mononuclear cells (PBMC) after T cell mitogen stimulation, suggesting impaired T cell function compared to stage 0 subjects. In the same study, stage 2 patients also produced lower levels of IL-6 after LPS stimulation, suggesting impaired antigen presenting cell function compared to stage 0 subjects (4).

More recently, Li et al. observed a striking loss of Treg cells in visceral adipose tissue of obese mice, driven by IFN-γ producing dendritic cells (30). The loss of circulating Treg cells has also been previously observed in humans in the context of obesity (31). Conversely, van der Weerd et al. found that morbidly obese subjects (BMI > 40) had higher absolute counts of Treg as well as naïve and memory T cells in PBMC compared to lean subjects (32). Higher proportions of CD4+ Th cells in PBMC were also observed in obese subjects (BMI > 35) compared to lean controls (33). In addition to obesity, factors such as hyperglycemia, dyslipidemia and insulin resistance have been shown to further modulate circulating T cell subtypes by increasing the ratio of Th1 to Th2 cells (34). Recent findings from our group also demonstrated that hyperglycemia in obesity and type 2 diabetes leads to an increased proportion of activated Th cells (expressing CD278), cytotoxic T cells as well as inflammatory monocytes (CD14+CRTh2+) in PBMC compared to individuals with obesity that are metabolically healthy (4). Hence, obesity and/or hyperglycemia not only affect immune cells response to stimuli, but also the proportions of circulating T cell subsets.

Luck et al. recently found that obesity also significantly impairs intestinal B cell homeostasis and functions in a manner that reduces immunoglobulin (Ig) A+ B cells and IgA producing plasma cells in mesenteric lymph nodes, and secretory IgA antibody concentrations in colon in DIO mice (35). IgA is known to defend mucosal surfaces against pathogen breaching (36). High-fat diet fed IgA deficient mice were also found to have increased intestinal permeability and levels of serum endotoxins compared to wild type controls (35). Therefore, we can speculate that the dysregulation of intestinal B cell homeostasis and IgA production in the context of obesity further contributes to local and systemic inflammation. In another study, splenic B cells from DIO mice poorly responded to CpG+anti IgM stimulation *ex vivo* compared to lean controls, suggesting that obesity also impairs peripheral B cell functions, particularly the ability to produce IgM and IgG antibodies. In the same study, B cells from PBMC of subjects with obesity produced less IL-6 upon *ex vivo* stimulation despite having elevated proportions of total B cells compared to lean subjects (37). DIO mice were also shown to have impaired antibody production upon influenza virus infection (38). Moreover, vaccinated individuals with obesity still displayed a remarkably higher risk of developing influenza compared to vaccinated lean subjects during the past influenza seasons (39). Thus, obesity seems to affect the proportion and functions of B cells not only at the local levels (i.e., adipose tissue and intestine) but also in circulation.

## The Interaction Between Obesity-Induced Dyslipidemia and Immune Dysfunction

Obesity induced chronic systemic inflammation impairs insulin signaling pathways via mechanisms such as serine phosphorylation of the insulin receptor and insulin receptor substrate-1 *via* the activation of IkB kinase, c-Jun N-terminal kinase and protein kinase C (40). At the same time, hypertriglyceridemia is thought to be a result of overproduction of TG-rich lipoproteins from the liver, and accelerated free fatty acids efflux from adipose tissue. On the other hand, overproduction of TG -rich chylomicrons from the intestine has also been observed in the context of obesity and insulin resistance (41). Obesity and insulin resistance are also thought to induce postprandial hypertriglyceridemia, which is a result of overproduction of chylomicrons from the intestine, overproduction of very-low density lipoprotein (VLDL) from the liver as well as limited endothelium lipoprotein lipase activity (42). Qin et al. demonstrated that TNF-α has a direct role in the overproduction of postprandial apoB48-containing lipoproteins (chylomicron) while upregulating II1-α, II6, and TNF-α gene expression and downregulating the gene expression of proteins related to insulin signaling in hamster enterocytes (43). Consequently, there appears to be evidence that inflammation contributes to dyslipidemia both in the fasting and postprandial states, and may in turn enhance systemic inflammation and impair the corresponding immune response.

Consistent with this finding, an *in vitro* study demonstrated that TG-rich lipoproteins activate human aortic endothelium cells to express vascular cell adhesion molecules (44). These adhesion molecules are in turn able to bind to circulating monocytes and facilitate their migration into the subendothelial space. Remnants of TG-rich lipoproteins are also small enough to penetrate the subendothelial space and offer additional sources of plaque-derived cholesterol (45). Internalized lipoprotein-cholesterol can subsequently activate numerous inflammatory signaling pathways and initiate the production of pro-inflammatory cytokines and chemokines (44). Also of note are studies that reported postprandial activation of neutrophils and monocytes while also increasing the total leukocyte count (reflected by expression of CD11b and CD66b) in human subjects after consumption of high fat meals (7, 46–48). Similar to postprandial TG dynamics, postprandial neutrophil counts are dramatically increased to a peak level in the first 2 h and reach a plateau after 4 h following a standard oral fat challenge (47). Stable isotope tracers, such as ^13^C-labeled palmitic acid and deuterium have been applied to modern nutritional studies in understanding endogenous lipid metabolism (49, 50). Taking advantage of this groundbreaking innovation of isotopic tracers in metabolic research, a study in which an oral fat load was conducted in eleven subjects using stable isotope tracers (^13^C-palmitate) demonstrated that postprandial leukocytes became enriched with meal-derived ^13^C-palmitate (7). This finding revealed a putative direct interaction between postprandial lipids and circulating leukocytes in that postprandial TG-rich lipoproteins may be directly phagocytosed or postprandial free fatty acid up-taken by leukocytes.

**Figure 1:**
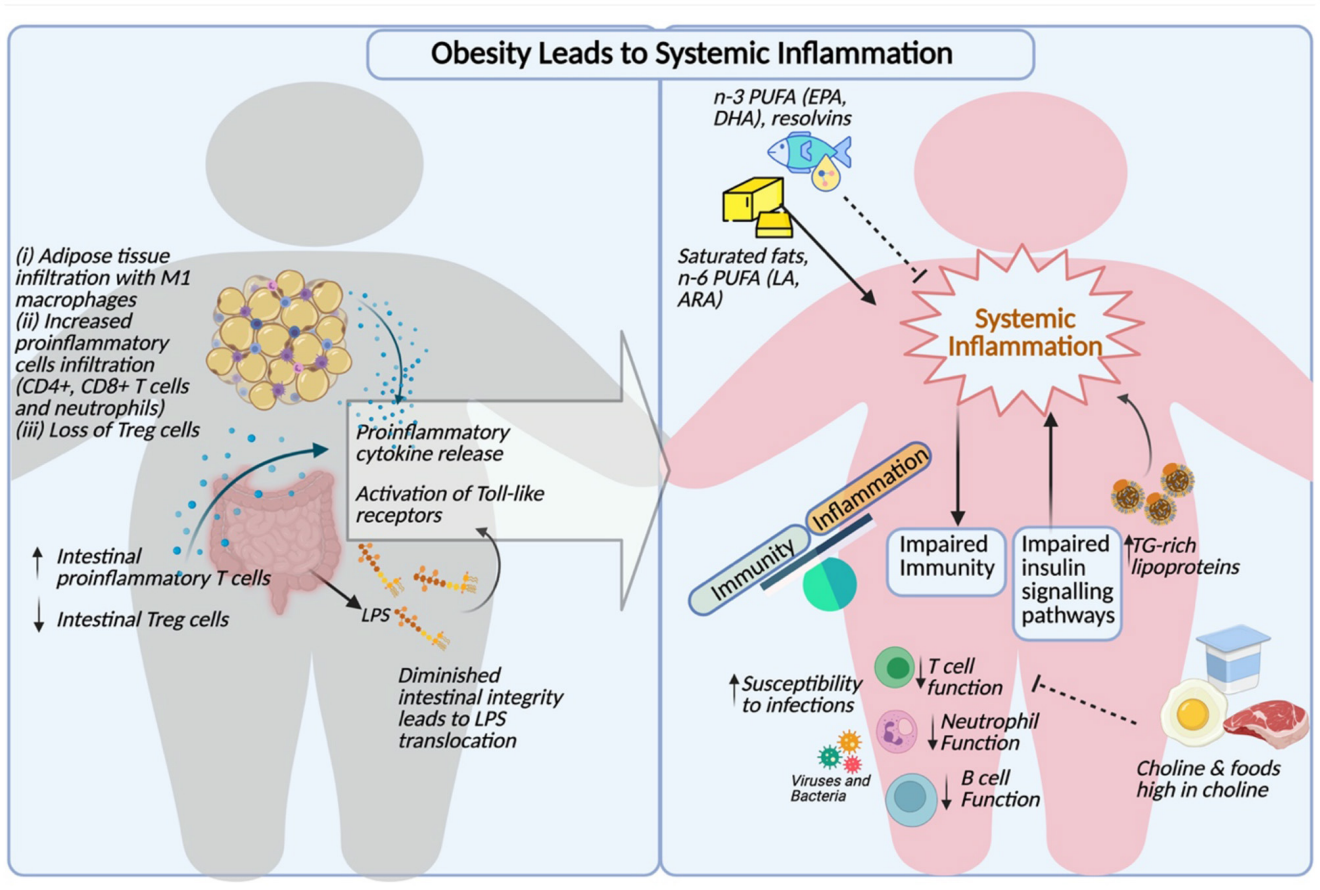
Comprehensive summary of obesity induced inflammation, dyslipidemia and impaired immunity (Created with BioRender.com).

These important findings suggest that hypertriglyceridemia can not only induce inflammation at a cellular level, but also serves as an activator of circulating leukocytes. The triglyceride-mediated immune cell activation may ultimately contribute to the progression of atherosclerosis and dysregulated immune function in the context of obesity. A comprehensive summary of the links between obesity, dyslipidemia and immune dysfunction is illustrated in Figure 1.

## Applicable Research Models of Obesity, Dyslipidemia and Immunology

### Rodents

Rodent models including rats and mice have long been applied to the field of research since the first development of a rat strain in 1920. In contrast to humans, high-density lipoprotein particles (HDL) were found to be the primary carrier of lipids in rats and mice. However, rats have been a good model for recapitulating the human findings, at least in certain types of immunological studies. For instance, gut-associated lymphoid tissue (GALT) is one of the most important secondary lymphoid organs that is linked to numerous metabolic complications. Rats and humans share substantial similarities in the GALT anatomy and they both have lymphocyte-filled villi (51). Given the development of modern genetic engineering, numerous rat and mouse models have been introduced to meet specific research needs. Some of the first phenotypic models used in this field include the fatty Zucker rat (52). The *fa/fa* homozygous Zucker rat strain spontaneously develops obesity, insulin resistance and hypertriglyceridemia (53). Later, another strain of Zucker rat, the Zucker diabetic fatty rat was developed (54). Other phenotypic rats of this kind have been developed such as the JCR:LA-cp rats. Homozygous JCR:LA-cp rats have impaired chylomicron and VLDL metabolism in the context of insulin resistance (53, 55). Ruth et al. have utilized the JCR:LA-cp rat model and showed that splenocytes of obese rats produced less IL-2 than lean controls after T cell mitogen stimulation, suggesting an impaired T cell function (56). However, spontaneous phenotypic rat models may be less relevant to understand the development of obesity in human and its nutritional modulation since they do not require diet-induced approaches. Lamas et al. was the first to demonstrate that cafeteria DIO Wistar rats had impaired T cell function compared to lean controls characterized by a lower IL-2 production from splenocytes after T cell mitogen stimulation (29). Sprague Dawley rats are another well-known wild-type model and often serve as the stock background for numerous genetic-engineered models (57). Recently, our lab demonstrated the benefits of supplementing rodent diets with different forms of choline, an essential nutrient, in Sprague Dawley rats, on the immune system (58–61).

Some spontaneous mutation models such as *ob/ob* and *db/db* mice have also been successful models to feature leptin deficiency and/or defective leptin binding and leading to obesity, dyslipidemia and other related commodities (53). *ob/ob* model is certainly one of the most used models in unraveling molecular basis of obesity-induced perturbations. Hsu et al. also found that *ob/ob* mice have an impaired pulmonary bacteria clearance after *Streptococcus pneumoniae* challenge, and increased death (62). Leptin plays a crucial role in modulating immune function, such as increasing T cell proliferation, and modulating expression of activation markers on both helper and cytotoxic T cells (63). Patients with obesity often display hyperleptinemia and leptin resistance, whereas this condition is certainly absent in *ob/ob* mice. These models are also monogenetic which is a rare condition in humans. Therefore, the DIO mouse model has emerged as a useful alternative. Luck et al. showed that DIO C57BL/6 mice have a shift to pro-inflammatory immune cells in intestinal lamina (3). This polarization of intestinal immune cells is characterized by an increased proportion of Th1 cells and cytotoxic T cells and a reduction in Treg cells (3). Amar et al. have also found that DIO mice respond poorly to *P.gingivalis* infection compared to the wild type lean control (64). However, careful consideration must also be taken when employing DIO mice models, as phytoestrogens are often absent in high fat diets but present in chow diets and known to impact rodent physiology (63). Therefore, when assessing the effect of diet/nutrients on the immune system using rodent models, special attention should be considered in carefully balancing the composition of chow/low-fat and high-fat diets. Moreover, IL-8 is a crucial chemokine for neutrophil recruitment during inflammation, however *IL8* gene is also absent in mice (65). Adaptive immunity such as T and B cell development in mice and Fc receptor expression were also found to be quite different from humans (66).

It is worth noting a variety of polygenic and even transgenic rodent models, such as GLUT4 glucose transporters mice, are also dominating the research and unraveling the molecular basis of obesity-induced cardiometabolic complications (67). More recently, CRISPR-Cas9 mediated leptin and leptin receptor knockout mouse models were also introduced (68). Despite a large body of evidence demonstrating the usefulness of employing rodent models in obesity-related research and using modern genetic engineering to introduce specific, attractive and sophisticated gene-knockout models, we must admit that these conditions are rare in humans. Additionally, differences in anatomy, physiology, lipid metabolism and immune systems between rodents and humans also needs to be taken into account when interpreting the findings from these studies.

### Large Animal Model-Swine

In contrast to rodent models, large animals such as pigs share tremendous similarities with humans in terms of anatomy, genetics and physiology. To highlight the similarities, pig-to-primate organ transplantation has been successfully achieved (69). Intriguingly, studies have demonstrated that intrauterine growth restriction pigs are more susceptible to insulin resistance and related metabolic complications later in life, making this a potentially useful model to study obesity-related complications in humans (70). The immune system composition of the pig also closely resembles that of humans, whereas mice only share about 10% similarity (69). For instance, 50 to 70% peripheral leukocytes are neutrophils in pigs which is quite similar to humans, and IL-8 has a direct ortholog in pigs for neutrophil recruitment (69). Circulating levels of IL-6 in pigs appear to be high and primarily originate from adipocytes, and dietary fatty acids were found to modulate endotoxin (LPS) levels and toll-like receptor 4 (TLR4) expression in swine adipose tissue (71). These findings highlight that pigs share similarities in immune response with humans and could be an alternative approach to study obesity-related immune dysfunction.

Currently, a number of swine models have been established to study obesity-related metabolic complications and pathologies, including the Yucutan miniature pig model and Ossabaw pigs (71–73). Yucutan miniature pigs have been shown to spontaneously develop impaired lipid and glucose metabolism (56). The Ossabaw pig model has also been described to develop criteria of metabolic syndrome including early insulin resistance, hypertriglyceridemia, hypertension, and visceral adiposity when fed a high fat diet (71). Recently, our own group has also established a swine model of insulin resistance using Duroc x Large White-Landrace low birth weight (LBW) piglets fed with high fat diet. The LBW high fat diet fed piglets developed signs of early insulin resistance, impaired postprandial TG metabolism and high hepatic bridging fibrosis after 6 weeks of intervention (74, 75).

Consistent with the findings that were found in rodent models and humans, recent work from our group has confirmed that high-fat diet induced insulin resistance in LBW swine exhibit impaired IL-2 production from PBMC after *ex vivo* mitogen stimulation, suggesting impaired immune function, particular to T cell function (76). Pawar et al. has also found increased expression of TNF-α in abdominal subcutaneous adipose tissue of obese pigs fed with high-fat/high-fructose diet, along with macrophage infiltration in both abdominal and pericardial adipose tissues, which paralleled the observations made in humans with obesity (77). The fact that these findings are consistent across different animal models as well as in humans further advances the concept that immune function is significantly impaired in the context of obesity and insulin resistance. It also highlights that swine may serve as a great intermediate between rodents and humans in studying obesity-related perturbations.

However, the swine model has major drawbacks such as being difficult to manage and maintain, and requiring specialized facilities. Routine and proper socializations with humans are also required to minimize pigs' stress and behavioral problems. Additionally, they can be very expensive to purchase. For instance, a 6 month-age miniature pig is ~1,000 US dollars (53). Similar to rodent models, a number of genetically modified diabetic pigs (such as CRISPR-Cas9 mediated) are also being introduced to meet specific research needs (78, 79); however, these strains are highly dedicated and can be even more expensive to purchase. Hence, swine strains that are originally maintained for food production may be the more economical and practical choice for large animal research in nutritional studies.

### Other Animal Models

In addition to the commonly used rodent models and swine models, a number of other species are also advancing the field of research. For instance, ferrets have been recognized as a useful intermediate model between rodent and non-human primate models in biomedical research, particularly for their use in researching infectious respiratory diseases (80, 81). Human pathogens are able to naturally infect ferrets and reproduce human diseases, highlighting the importance of this model in immunological research in order to recapitulate human findings. DIO (National Institute of Health, USA ongoing project) and CRISPR-Cas9 genetic modified ferret models are also gradually introduced (82). However, this model is currently very rare in nutritional studies. Limitations such as lack of commercial antibodies is one of the main drawbacks. Rabbit is a species that can develop all components of the metabolic syndrome similarly to humans and are easy to handle and cost-effective (83). DIO rabbit models have also been widely used in understanding obesity-related cardiometabolic complications (84, 85). They are also extremely sensitive to cholesterol and therefore, more often used as a pre-clinical model for atherosclerosis research (53). Rabbits have also been used in a series of immunological studies and became major sources for the production of mono- and polyclonal antibodies (86). Finally, non-human primates such as monkeys and rhesus are no doubt the most ideal model since they are highly relevant to human anatomy and physiology, are omnivorous and able to develop metabolic complications with aging (53, 67). Several DIO non-human primate models such as a high-fat high-sugar diet fed baboon model, a high-fat diet fed marmoset model as well as a Japanese macaque model were also developed to mimic the phenotype of obesity-related complications in humans (87). However, major drawbacks of non-human primates are lack of approved facilities, long life span and risk of transmitting infectious viruses to humans.

## Nutritional Strategies to Improve Immune Dysfunction in the Context of Obesity

One of the first lines of intervention to improve cardiometabolic health and immune function is certainly weight loss and physical activity. Physically active subjects were found to have lower circulating CRP, CD14+CD16+ monocytes and LPS-induced TNF-α production from whole blood compared to physically inactive individuals (88). Exercise was also found to modulate adipose tissue macrophage infiltration, in a manner that downregulated MCP-1 following a moderate-intensity excise protocol (89). In addition to physical exercise, a number of pharmacological therapies have also been developed with promising potential to improve obesity and type-2 diabetes associated immune dysfunction, such as etanercept (TNF-α blockade) (90).

Besides genetic and environmental factors, obesity is often caused by excessive energy intake combined with reduced energy expenditure, reinforcing the fact that adequate and appropriate nutrition is required. Accumulating evidence has indicated that consumption of a high-fat meal has a direct role in promoting postprandial inflammation and endotoxemia in humans (91). However, nutrients have diverse roles and often act differently at modulating immune function. Saturated fatty acids (SFA) were found to be pro-inflammatory and share similar structural components of gut bacteria endotoxins (LPS). Among all SFA, lauric acid is thought to exert the most pro-inflammatory properties, whereas myristic and stearic acids tend to exert the least (92). Polyunsaturated fatty acids (PUFA) of the omega-6 family (linoleic acid, LA; arachidonic acid, ARA) are generally considered pro-inflammatory whereas omega-3 (eicosapentaenoic acid, EPA; docosahexaenoic acid, DHA) are considered anti-inflammatory. This is due to the fact that their derivate metabolites (i.e. eicosanoids and resolvins) primarily exert pro/anti-inflammatory activities, respectively. However, this is an oversimplification since some of ARA derived eicosanoids, such as lipoxin 4, can also be anti-inflammatory and not all EPA derived metabolites are anti-inflammatory (93). ARA derived lipoxins and epi-lipoxins were also found to promote resolution of inflammation by regulating the life cycle of immune cells including neutrophils and macrophages (94). The recent work conducted at Texas Tech University by Davis and colleagues demonstrated that EPA ameliorated gene expression of inflammatory markers in DIO B6 mice, along with reducing macrophage infiltration into epididymal white adipose tissue, in a dose-dependent manner (95). Additionally, other bioactive fatty acids such as ruminant-derived trans fats have been shown to be beneficial to immune function including actions on IL-2 and TNF-α production after mitogen stimulation (56, 96).

### Micronutrients and Immune Function

Selenium, a trace element, has critical functional roles in a range of physiological responses. Selenium deficiency has also been found to be associated with a higher risk of cancer and CVD (97). Selenium supplementation was found to increase T cell proliferation and IFN-γ production in adults with marginal selenium status (98). The key roles of a number of other micronutrients including Vitamin A, B, C, D and E, and trace minerals in supporting immune function are also widely appreciated and reviewed elsewhere (99). In recent years, a better understanding of the role of dietary choline at modulating immune function have also emerged. Choline serves as the precursor for functional and structural components of cell membranes and critical to a number of molecules including neurotransmitters and lipoproteins. Dietary forms of choline can be divided into water-soluble forms [i.e., free choline (FC), phosphocholine (PCho) and glycerophosphocholine (GPC)] and lipid-soluble forms [phosphatidylcholine (PC) and sphingomyelin (SM)] (100). We have previously demonstrated that feeding choline in the form of PC to Sprague-Dawley dams during lactation improves IL-2, IL-6 and IFN-γ production from splenocytes after *ex vivo* mitogen stimulation in their offspring pups, highlighting the critical role of choline in offspring's immune system development (58). Consistent with this, recent work from our own group has also found that feeding buttermilk-derived choline forms (37% PC, 34% SM, 17% GPC) to Sprague Dawley dams during pregnancy and lactation led to a higher IL-2, TNF-α and IFN-γ production from splenocytes after mitogen stimulation compared to the control diet in lactating dams (61), suggesting an improved immune function during lactation. Later, we have also demonstrated that the lipid soluble form of choline (especially PC and SM), in the suckling and weaning diet, improved immune function early in life in Sprague Dawley dams' offspring (60). These pre-clinical findings remain to be confirmed in humans. Nevertheless, emerging evidence is highlighting the promising role of choline and its different forms, along with other important micronutrients, in supporting the function of the immune system in different life stages in the context of obesity.

### Dairy Intake, Insulin Resistance and Immune Function

Biomarkers for dairy fat intake have been found to inversely associate with variables of insulin resistance and incidence of type-2 diabetes in a pooled analysis of 12 cohort studies (101). However, high SFA found in dairy fat has traditionally been a concern to many health organizations, including Health Canada, who revised the Canada's food guide in 2019 which no longer recommends 2–3 servings of dairy products/day. Accumulating clinical evidence has shown that dairy consumption has no impact on inflammation particularly in subjects with obesity or are overweight (102, 103). Another recent systematic review has concluded that consumption of various forms of dairy products may have either favorable or neutral effects on several cardiometabolic risk factors, including the lipid profile and systemic inflammation (104). Interestingly, low-fat yogurt consumption was also found to attenuate postprandial inflammation by decreasing the plasma LPS binding protein to CD14 ratio and IL-6 concentrations (105). The recent preliminary findings from our group found that consumption of 3 servings/day of low-fat dairy products normalized IL-2 production from PBMC in a swine model of insulin resistance, suggesting improved T cell function as compared to controls (76). The immune-protective benefits associated with dairy intake is also likely due to the presence of dietary choline. Our group has assessed the amount of choline in a variety of commercial dairy products in Canada and reported that there is a negative correlation between the fat content in dairy and choline content suggesting that low-fat dairy foods generally contain higher amount of choline (106). Moreover, a cohort study has reported that the main sources of dietary choline consumed by the population come from dairy, meat and egg (107). Therefore, dairy is an important source of choline and especially lipid soluble forms of choline that could potentially beneficially affect immune function in the context of obesity.

## Conclusion

The rising concern in the global prevalence of obesity and its associated health consequences has drawn substantial attention from scientists in the areas of physiology, immunology and nutrition. Although numerous underlying mechanisms that link obesity and immunity are still poorly understood, we have highlighted a number of obesity-related cardiometabolic and immune dysfunctions that underpin the obesogenic etiology. Research models that are driving this field of research range from small animal models of rodents to emerging large swine animal models. In the past few years, numerous pharmacological and nutritional strategies to improve immune dysfunction in the context of obesity have been developed to meet specific needs. Despite this, ongoing research is required in order to translate these findings into tenable advances in the treatment or risk management of obesity in humans.

## Author Contributions

CR and SP obtained funding for this study. YS wrote the manuscript under the supervision of CR and SP. CR has primary responsibility for final content. All authors have read and approved the final manuscript. All authors contributed to the article and approved the submitted version.

## Funding

This study was supported by grants from Dairy Farmers of Canada (RES0042193) and the Natural Sciences and Engineering Research Council of Canada (RES0038933). YS is a recipient of a Ph.D. scholarship from China Scholarship Council (201807980001).

## Conflict of Interest

The authors declare that the research was conducted in the absence of any commercial or financial relationships that could be construed as a potential conflict of interest.

## Publisher's Note

All claims expressed in this article are solely those of the authors and do not necessarily represent those of their affiliated organizations, or those of the publisher, the editors and the reviewers. Any product that may be evaluated in this article, or claim that may be made by its manufacturer, is not guaranteed or endorsed by the publisher.
