# Corrigendum: The interplay of obesity, dyslipidemia and immune dysfunction: a brief overview on pathophysiology, animal models, and nutritional modulation

**DOI:** 10.3389/fnut.2023.1304102

**Published:** 2023-10-11

**Authors:** Yongbo She, Rabban Mangat, Sue Tsai, Spencer D. Proctor, Caroline Richard

**Affiliations:** ^1^Division of Human Nutrition, Department of Agricultural, Food and Nutritional Science, University of Alberta, Edmonton, AB, Canada; ^2^Metabolic and Cardiovascular Diseases Laboratory, Department of Agricultural, Food and Nutritional Science, University of Alberta, Edmonton, AB, Canada; ^3^Department of Medical Microbiology and Immunology, University of Alberta, Edmonton, AB, Canada

**Keywords:** obesity, insulin resistance, dyslipidemia, immune function, nutrition

In the published article, there was an error in the **Acknowledgements** statement. Support received by CR from the Canada Research Chair program was omitted from the statement.

The corrected Acknowledgements statement appears below.

## Acknowledgments

CR was supported by the Canada Research Chair program (CRC-2018-00081).

The authors apologize for this error and state that this does not change the scientific conclusions of the article in any way. The original article has been updated.

